# A prospective comparison of the diagnostic performance of a high b-value diffusion-weighted image-guided score versus PI-RADS v2.1 for transition zone prostate cancer

**DOI:** 10.3389/fonc.2026.1764833

**Published:** 2026-05-08

**Authors:** Kexin Li, Tianquan Xu, Xin Yan, Shaonan Mi, Miaomiao Jiang, Lu Chen, Fu Kuang

**Affiliations:** Department of Radiology, The Second Affiliated Hospital of Harbin Medical University, Harbin, Heilongjiang, China

**Keywords:** diffusion magnetic resonance imaging, PI-RADS v2.1, prostate biopsy, prostate cancer, transition zone prostate cancer

## Abstract

**Objective:**

The objective of this study is to evaluate the diagnostic accuracy of transition zone (TZ) scoring guided by diffusion-weighted imaging (DWI) with different b-values versus TZ lesion scoring under the current PI-RADS v2.1 standard for diagnosing transition zone prostate cancer (TZPCa).

**Methods:**

This prospective study enrolled 345 men with suspected prostate cancer (PCa). Following standardized multi-parametric magnetic resonance imaging (mpMRI), all patients underwent cognitive fusion-guided targeted biopsy and systematic biopsy, ultimately enrolling 50 patients with TZPCa and 121 patients with benign prostatic hyperplasia (BPH). DWI sequences were acquired with b-values of 50, 800, 1500, 2500, and 3000 s/mm². Three contrast groups were established for comparison with conventional PI-RADS v2.1 TZ scoring: DWI-TZ group: b=50, 800, 1500 s/mm²; h-DWI-TZ group: b=50, 800, 2500 s/mm²; uh-DWI-TZ group: b=50, 800, 3000 s/mm². Scoring procedure: Each radiologist first scored TZ lesions using standard b-values (50, 800, 1500) according to PI-RADS v2.1 criteria. After a 4-week interval, DWI-guided TZ scoring was performed on images from the three distinct b-value groups. The procedure for each group was identical: first, identify suspicious lesions on DWI/ADC images; then correlate these with T2WI images; finally, score TZ lesions according to PI-RADS v2.1 criteria. All scoring was performed independently by two radiologists.

**Results:**

When PI-RADS ≥ 3 was employed as the positive criterion, both the high b-value (h-DWI-TZ) and ultra-high b-value (uh-DWI-TZ) sequences exhibited significantly higher diagnostic sensitivity (0.855 and 0.823, respectively) and specificity (both 0.863) in comparison to conventional DWI sequences (sensitivity 0.677, specificity 0.630; p < 0.05 for both). Conversely, the standard high b-value (DWI-TZ) sequence demonstrated no statistically significant difference from the conventional sequence. When the threshold was elevated to PI-RADS score ≥4, the h-DWI-TZ sequence exhibited significantly superior specificity (0.967 vs. 0.874) and positive predictive value (PPV, 0.902 vs. 0.630) in comparison to the conventional sequence (both p<0.001). In addition, the h-DWI-TZ sequence exhibited markedly superior interobserver agreement (κ-value: 0.689 vs. 0.272, p<0.001).

**Conclusions:**

Diffusion-weighted imaging with high and ultra-high b-values guided TZ scoring enhances diagnostic accuracy for TZPCa.

## 1 Introduction

Prostate cancer (PCa) is the second most common malignant tumour in men worldwide (1, 2), with 30% of cases occurring in the transition zone(TZ) (3). Both benign prostatic hyperplasia (BPH) and TZPCa present as low-signal nodules in the TZ on T2-weighted images (T2WI) (4), with their morphological similarity posing the primary challenge for differential diagnosis (5–7).

Multi-parametric magnetic resonance imaging (mpMRI) based on the PI-RADS v2.1 criteria serves as the primary modality for detecting TZPCa and performing its radiological risk stratification (8). Even though the PI-RADS v2.1 scoring system has become the clinical standard (8, 9), it retains certain limitations when evaluating TZ lesions. The official guidelines explicitly state that T2WI serves as the primary basis for TZ assessment, with diffusion-weighted imaging (DWI) permitted to supplement grading (8, 9). Nevertheless, its diagnostic specificity for TZPCa remains suboptimal, and inter-reader agreement demonstrates only moderate consistency (10–13). DWI is crucial for lesion classification (10–16), and the DWI-guided TZ scoring method proposed by Lee et al. (17) can improve the detection rate of TZ lesions. However, the b-value used (1400 s/mm²) is only the minimum threshold required by PI-RADS v2.1. This b-value is not optimal for suppressing background signals from benign lesions (such as BPH or inflammation), which may compromise clear visualization of lesions (18). More significantly, apparent diffusion coefficient (ADC) values calculated from a single b-value exhibit considerable overlap in range between high-grade (high Gleason score) and low-grade PCa (19, 20). It also fails to reduce interference from high central glandular signals. Its impact on improving sensitivity and negative predictive value is also limited. Higher b-values can enhance the contrast of malignant lesions and suppress false diffusion (21–25); however, their systemic diagnostic efficacy for TZPCa remains unclear.

This study aims to compare the diagnostic efficacy of transition zone (TZ) scores guided by DWI at different b-values with the PI-RADS v2.1 scoring system (25). It further analyzes the diagnostic performance variations of DWI-guided TZ scores under different b-value conditions to investigate whether increasing the b-value enhances the detection rate of TZPCa.

## 2 Materials and methods

### 2.1 Study design

This prospective study was approved by the Ethics Committee (Approval No: KY2024-246). All participants received comprehensive information about the study before enrollment. The study was conducted in accordance with the principles outlined in the Declaration of Helsinki.

**Figure 1:**
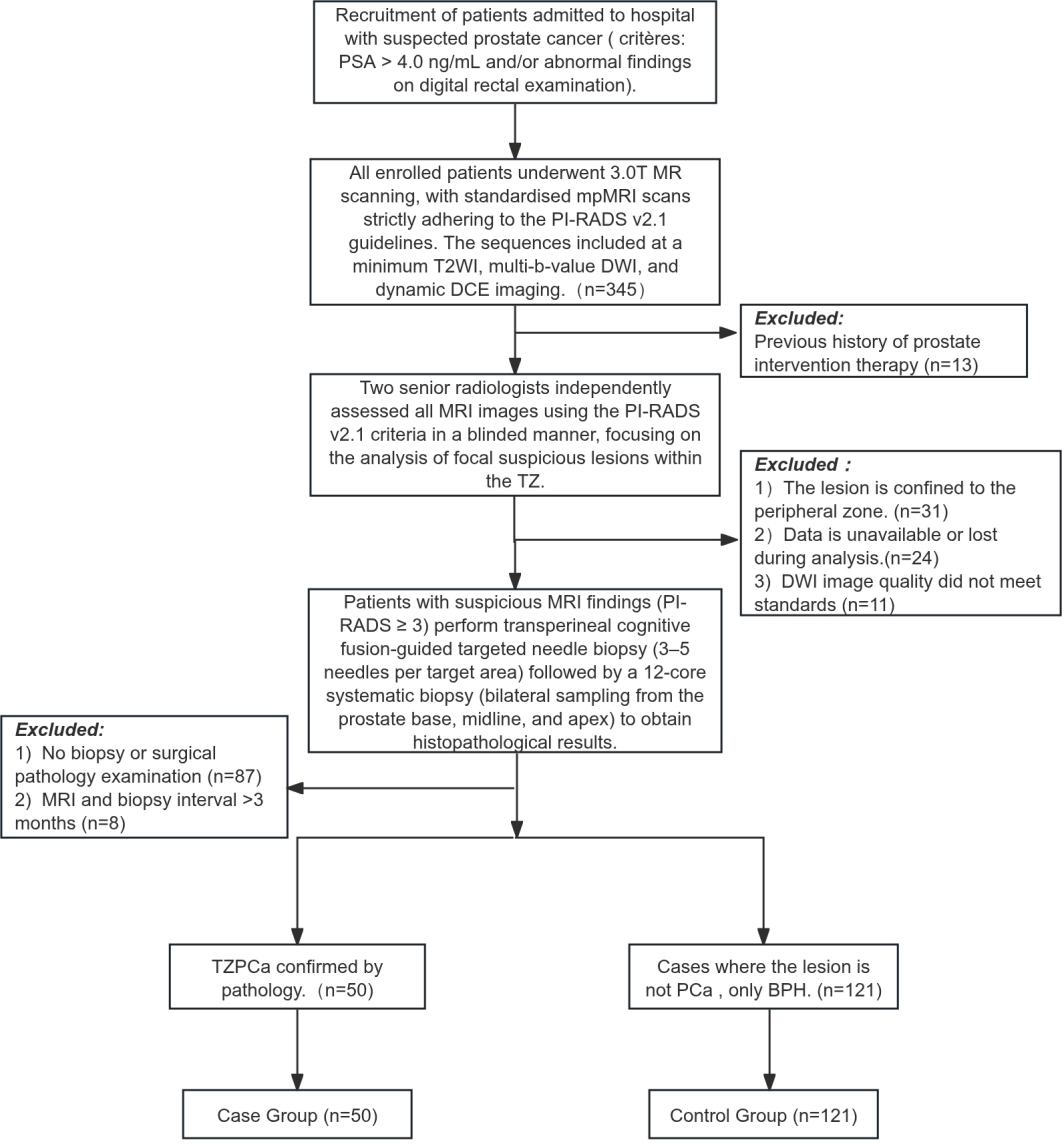
Patient flow chart.

In the period from October 2024 to September 2025, the present study prospectively enrolled 345 male patients suspected of having PCa based on abnormal prostate-specific antigen (PSA) levels (>4 ng/mL) and/or suspicious findings on digital rectal examination (DRE). All patients underwent a standardised multiparametric MRI examination. Exclusion criteria were: 1) Previous history of prostate intervention therapy (n=13), including surgery, radiotherapy, chemotherapy, or hormone therapy, to prevent treatment-related morphological changes from interfering with lesion assessment; 2) The lesion is confined to the PZ (n=31); 3) Data is unavailable or lost during analysis (n=24); 4) DWI image quality did not meet standards (n=11): due to artefacts or defects that did not meet analysis requirements (26); 5) No biopsy or surgical pathology examination (n=87); 6) MRI and biopsy interval >3 months (n=8): to minimise assessment bias caused by new lesions (27). Ultimately, 50 patients with pathologically confirmed TZPCa were included in the case group. **Figure 1** details the patient inclusion process.

This study evaluated the effectiveness of DWI-guided TZ scoring for TZPCa across various b-value combinations. It employed a lesion-level analysis strategy rather than a patient-level one. The final analysis included 197 lesions (from 171 patients), with 62 true-positive lesions in the case group and 135 false-positive lesions in the control group. The case group comprised 10 patients with multifocal TZPCa, encompassing a total of 62 true-positive lesions. The 135 false-positive TZ lesions in the control group were also included in the statistical analysis. False-positive lesions were defined as those reported as positive by at least one radiologist (PI-RADS ≥ 3) but confirmed pathologically as BPH or Granulomatous Prostatitis (GP).

### 2.2 Data acquisition and postprocessing

Comprehensive patient data—including age at diagnosis, serum prostate-specific antigen (PSA) levels, Gleason score (GS), and number of TZ cancer foci—were systematically collected. Definitive histopathological confirmation served as the diagnostic gold standard for all TZPCa cases (**Table 1**).

**Table 1:** Patient demographics.

Variablaes	Value
Case group(n=50)	——
Age, year (Mean, Range)	62.0(51-74)
PSA, ng/mL((Mean ± SD)	19.82 ± 7.65
Control Group (n = 121)	——
Age, year (Mean, Range)	60.3(48-71)
PSA, ng/mL (Mean ± SD)	12.51 ± 7.04
Number of TZPCa per Case Group patients (n = 50)	——
1	40
2	8
3 or more	2
Lesions (n = 62)	——
Gleason Score (GS)	——
6	16(25.8%)
7(3 + 4 = 7)	9(14.5%)
7(4 + 3 = 7)	12(19.4%)
8(4 + 4 = 8)	10(16.1%)
9(4 + 5 = 9)	11(17.7%)
9(5 + 4 = 9)	4(6.5%)
PI-RADS v2.1 score distribution for lesions in the control group (radiologists with 5 or 11 years of experience)	——
1	13/29
2	58/67
3	42/27
4	16/8
5	6/4

All patients underwent a fast that lasted three hours and were guaranteed to have complete rectal evacuation. The MRI examinations were performed on a 3T scanner (uMR 790, United Imaging Healthcare, Shanghai, China). The patient was imaged using an abdominal coil with 12 channels and a spinal coil with 32 channels. The mpMRI protocol encompassed the following sequences: axial T1-weighted images (T1WI), axial and coronal T2-weighted images (T2WI), axial dynamic contrast-enhanced (DCE) sequences, and axial DWI sequences (b = 50, 800, 1500, 2500, 3000 s/mm²). ADC maps were automatically generated at the workstation. A synopsis of salient protocol particulars is delineated in **Table 2**.

**Table 2:** Sequence parameters for multiparametric MRI of the prostate.

Sequence parameter	T2WI TSE	DWI	DCE
Imaging plane	Axial	Axial	Axial
TR/TE (ms)	7257/95	5000/69	4.0/1.44
Slice thickness	3	3	2
Interslice gap	0	0	0
Field of view (FOV)(mm)	200×200	300×300	350×317
Matrix size	352×277	80×80	288×230
In-plane resolution(mm²)	0.57×0.72	3.75×3.75	1.22×1.38
Parallel imaging factor	1.4	2.0	2.1
Acquisition time	2min40s	6min40s	3min05s
b value(s/mm^2^)	——	0; 800; 1500; 2500; 3000	——

### 2.3 Data on MRI-pathology matching

This study was conducted through the collaborative efforts of experienced radiologists, sonographers, and pathologists. The workflow encompassed the following stages:

1. MRI Examination and Assessment: Before biopsy, multiparametric MRI was performed to assess prostate size and morphology, thereby identifying and localizing suspicious lesions within the TZ.
2. Biopsy Procedure: Patients were positioned in the lithotomy position. The procedure was performed under dual local anesthesia, comprising skin infiltration anesthesia and a prostatic capsular nerve block. Initially, transperineal cognitive fusion-guided targeted needle placement was conducted, involving the insertion of three to five needles per target area. Subsequently, a 12-core systematic biopsy was performed, entailing bilateral sampling from the base, midportion, and apex of the prostate, to assess tumour burden and identify MRI-silent lesions comprehensively.

### 2.4 Qualitative analysis

This study involved a senior radiologist initially classifying mpMRI imaging data from the patient and control groups: Group A included axial T1WI, axial and coronal T2WI, and DWI (b=50, 800, 1500 s/mm² images); Group B included axial T1WI, axial and coronal T2WI, and DWI (b=50, 800, 2500 s/mm² images); Group C included axial T1WI, axial and coronal T2WI, and DWI (b=50, 800, 3000 s/mm²) images. For clarity, these three groups were assigned distinct labels: standard DWI-TZ (Group A, b=1500 s/mm²), high b-value DWI-TZ (h-DWI-TZ, Group B, b=2500 s/mm²), and ultra-high b-value DWI-TZ (uh-DWI-TZ, Group C, b=3000 s/mm²). Before distribution, all patients’ personal information was anonymised and fully randomised. Image analysis was performed and documented independently by two radiologists with differing levels of experience (Reader 1: 5 years of experience; Reader 2: 11 years of experience). They evaluated only TZ lesions and were unaware of any patient clinical information. The analysis proceeded in four stages: Initial Interpretation: Both physicians used Group A imaging data to score TZ lesions strictly according to the PI-RADS v2.1 criteria. Second reading (4 weeks later): Both physicians again used Group A data but applied the “DWI-guided TZ scoring method.” Specifically, they first identified suspicious lesions on DWI/ADC images with b-values of 50, 800, and 1500 s/mm², then correlated these with T2WI images, and finally scored them according to the PI-RADS v2.1 criteria. Third Interpretation (4 weeks later): Two physicians used Group B data, applying the same “DWI-guided TZ scoring method.” They first identified lesions on DWI/ADC images with b-values of 50, 800, and 2500 s/mm², then correlated these with T2WI for scoring. Fourth Interpretation (4 weeks later): Two physicians used Group C data, continuing the “DWI-guided TZ scoring method.” They first identified lesions on DWI/ADC images with b-values of 50, 800, and 3000 s/mm², then correlated with T2WI for scoring. For Interpretations 2-4, a specific scoring rule was applied: if DWI scored 4 but T2WI scored 3, the final PI-RADS classification was assigned a score of 3. Although standard PI-RADS v2.1 criteria for the TZ generally maintain a score of 4 when DWI = 4, we applied this modified rule based on Lee et al. (17) to test their proposed DWI-guided scoring system. If a lesion was not detected on DWI/ADC images, it was not scored even if visible on T2WI (a DWI-guided exclusion rule adapted from Lee et al. (17) to validate their algorithm). The number of lesions excluded based on this rule is detailed in the Results section. The second and subsequent interpretation methods were collectively termed the “DWI-guided TZ scoring method” to distinguish them from the initial conventional PI-RADS v2.1 analysis.

Although this approach deviates from the standard PI-RADS v2.1 protocol (where TZ assessment primarily relies on T2WI), it aims to validate the hypothesis that higher b-value DWI-TZ scores enhance diagnostic accuracy for TZPCa. This would facilitate identification of TZ malignancies exhibiting abnormalities on T2WI but without prominent functional imaging abnormalities, thereby optimising clinical risk stratification. All evaluations were performed on a dedicated workstation, with lesion localisation and measurement parameters documented. Intra-reader variability was assessed using intraclass correlation coefficients and Kappa statistics.

### 2.5 Statistical analysis

Differences in age and prostate-specific antigen (PSA) levels between patient groups were analyzed using the Mann-Whitney U test (non-parametric continuous data comparison) (28). For PI-RADS v2.1 scoring, two positivity thresholds (≥ three and ≥4) were evaluated. Inter-reader agreement between two radiologists was quantified via Cohen’s Kappa coefficient (κ) for dichotomous variables, with agreement strength interpreted per Landis and Koch criteria (29).

Given the lesion-level analysis and the possibility of multiple lesions per patient, all diagnostic performance metrics (including sensitivity, specificity, positive and negative predictive values, and the F1-score) were calculated using generalized estimating equations (GEE) to account for within-subject correlation and avoid overestimation of statistical significance due to clustering (30). In the GEE models, the patient ID was specified as the clustering variable, and an exchangeable correlation structure was used, as it is a common and parsimonious choice for modeling within-cluster correlation in this context.

All analyses were performed in R 4.1.2 (R Foundation for Statistical Computing, Vienna, Austria) and Python 3.12.3 (Python Software Foundation). In R, generalized estimating equations (GEE) were fitted using the *geepack* package, and inter-rater agreement (Cohen’s kappa coefficient) was assessed using the *irr* package. In Python, the statsmodels library was utilized for statistical modeling, and the scikit-learn library was employed for metric calculations. Statistical significance was defined as a two-tailed p< 0.05.

## 3 Results

### 3.1 Patient characteristics

The mean ages of the case group and control group were 62 years and 60 years, respectively. There was no statistically significant difference in age between the two groups (p = 0.943). The case group showed numerically higher serum PSA levels (19.82 ± 7.65 ng/mL vs. 12.51 ± 7.04 ng/mL), although this difference did not reach statistical significance (p = 0.174). Among the 50 patients in the case group, 10 had multifocal TZPCa (**Table 1**).

### 3.2 Lesion-based agreement between observers

Comparing with conventional PI-RADS v2.1 scoring, the three DWI-guided TZ scoring methods with different b-values demonstrated superior interobserver agreement (Cohen’s kappa) under various positive criteria: 1) Using a score ≥3 as the positive criterion: The κ values for DWI-TZ, h-DWI-TZ, and uh-DWI-TZ were 0.450, 0.689, and 0.664, respectively, all higher than the conventional method’s 0.272. 2) Using a score ≥4 as the positive criterion: The κ values for the three groups of b-value-guided TZ scoring methods were 0.544, 0.684, and 0.650, respectively, all higher than the conventional method’s 0.368. Results indicate that three DWI-guided TZ scoring methods enhance diagnostic consistency (31) (**Tables 3**, **4**).

**Table 3:** Cohen’s κ and F1 score with a PI-RADS v2.1 threshold of ≥3.

Method	κ-value	95% CI	p-value	F1 score
Conventional	0.272	0.175-0.368	p = 0.0017	0.545
DWI—TZ	0.450	0.357-0.543	p < 0.0001	0.643
hDWI—TZ	0.689	0.613-0.765	p < 0.0001	0.794
uhDWI—TZ	0.664	0.585-0.743	p < 0.0001	0.776

**Table 4:** Cohen’s κ and F1 score with a PI-RADS v2.1 threshold of ≥4.

Method	κ-value	95% CI	p-value	F1 score
Conventional	0.368	0.260-0.475	p < 0.0001	0.537
DWI—TZ	0.544	0.446-0.641	p < 0.0001	0.636
hDWI—TZ	0.684	0.602-0.766	p < 0.0001	0.769
uhDWI—TZ	0.650	0.564-0.736	p < 0.0001	0.742

Based on the DWI-guided scoring strategy, lesions not visible on DWI/ADC were excluded from analysis. This resulted in the exclusion of a limited number of lesions: for senior readers, 11 lesions in h-DWI-TZ, 10 in uh-DWI-TZ, and 4 in DWI-TZ; for less experienced readers, 7 lesions in h-DWI-TZ, 9 in uh-DWI-TZ, and 1 in DWI-TZ.

### 3.3 TZ scoring performance with different DWI b-values

**Figure 2:**
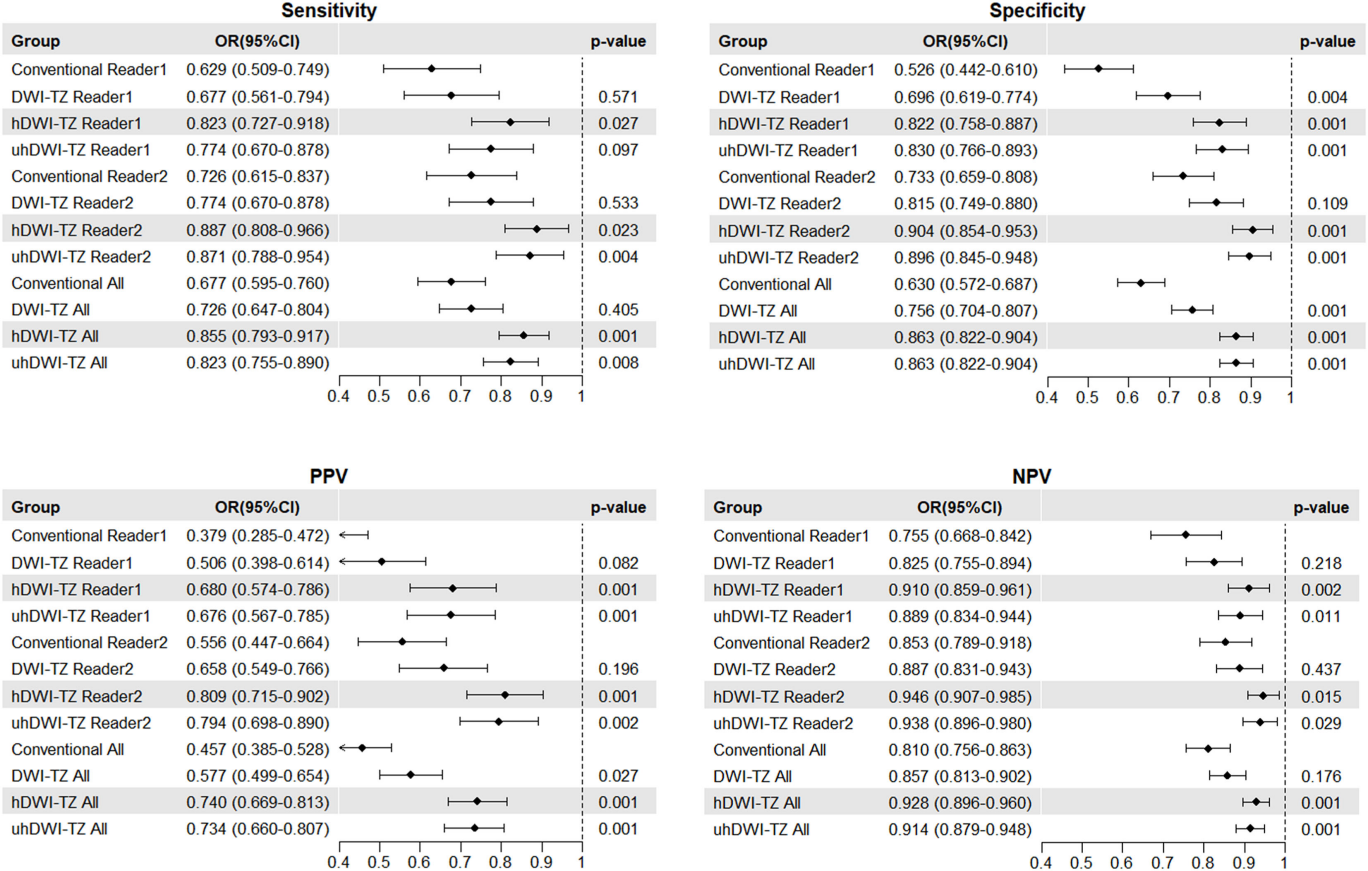
Diagnostic performance of conventional and three different B-value diffusion-weighted imaging-guided TZ scores for PI-RADS category 3 or greater positive lesions.

**Figure 3:**
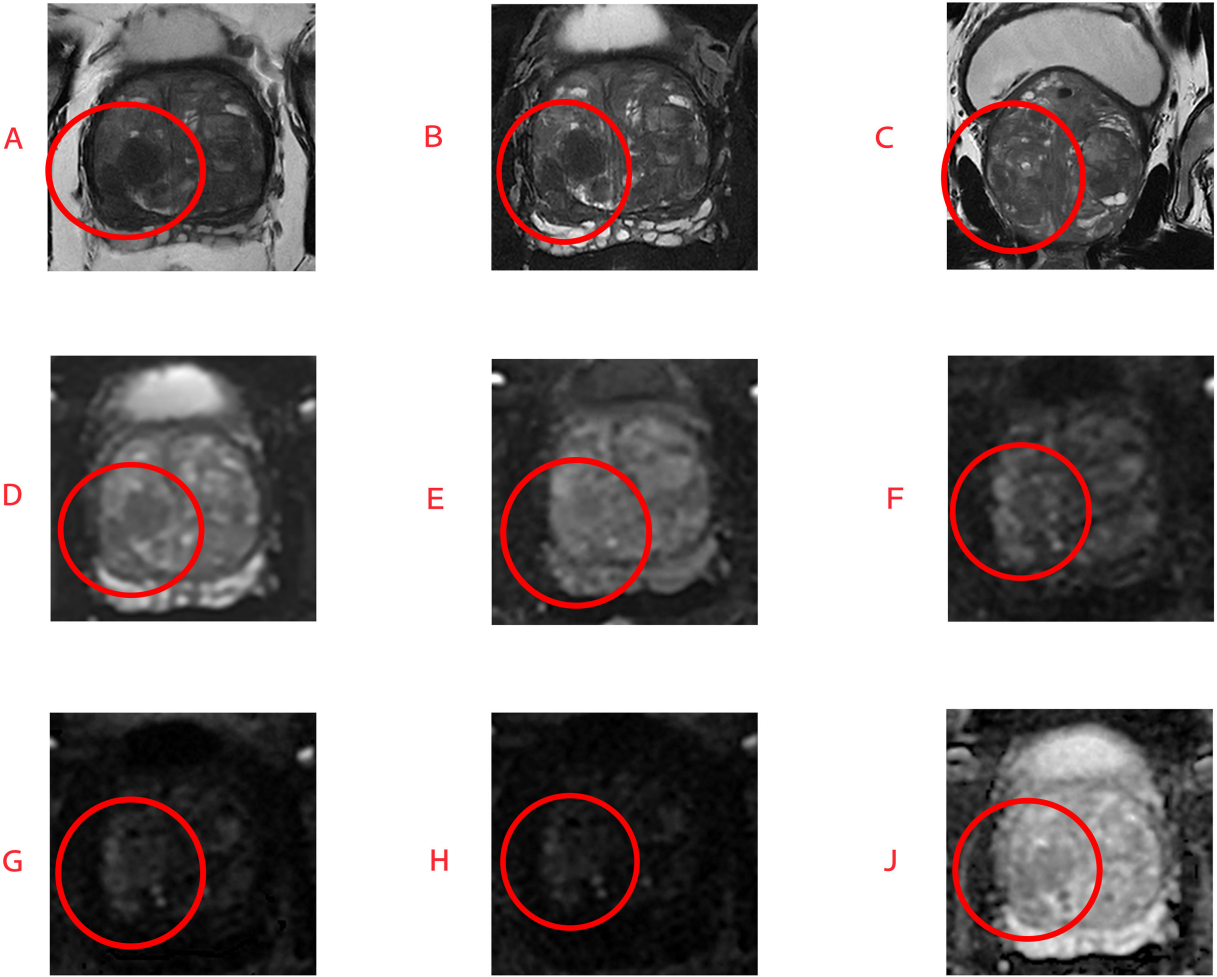
This is magnetic resonance imaging of a 60-year-old patient with benign prostatic hyperplasia. T2-weighted images **(A–C)** reveal a poorly defined low-signal lesion in the anterior transition zone; DWI sequences at b=0 **(D)** and b=800 **(E)** are also shown, and this lesion shows slightly increased signal intensity on the DWI sequence at b=1500 **(F)**, while no significant signal enhancement is observed on sequences at b=2500 **(G)** and b=3000 **(H)**. The ADC map is shown in **(J)**. Subsequent biopsy revealed no evidence of prostate cancer, further indicating the diagnostic value of high b-value DWI for lesions in the prostate TZ, transition zone.

The study findings indicate that when using a PI-RADS score ≥3 as the positive threshold, the specificity of TZ scores guided by DWI-TZ, h-DWI-TZ, and uh-DWI-TZ was significantly higher than that of conventional scoring (0.756, 0.863, and 0.863, respectively, compared to 0.630; p<0.001). Additionally, h-DWI-TZ and uh-DWI-TZ-guided TZ scoring demonstrated significantly superior sensitivity and NPV compared to conventional PI-RADS v2.1 scoring. In contrast, DWI-TZ scoring showed no significant difference in sensitivity, PPV, or NPV relative to traditional scoring. Relevant figures are shown in **Figures 2**, **3**. Although both h-DWI-TZ (b=2500) and uh-DWI-TZ (b=3000) demonstrated superior performance compared to standard DWI-TZ, direct comparison between h-DWI-TZ and uh-DWI-TZ revealed no statistically significant differences in diagnostic performance for either reader (all p>0.05).

**Figure 4:**
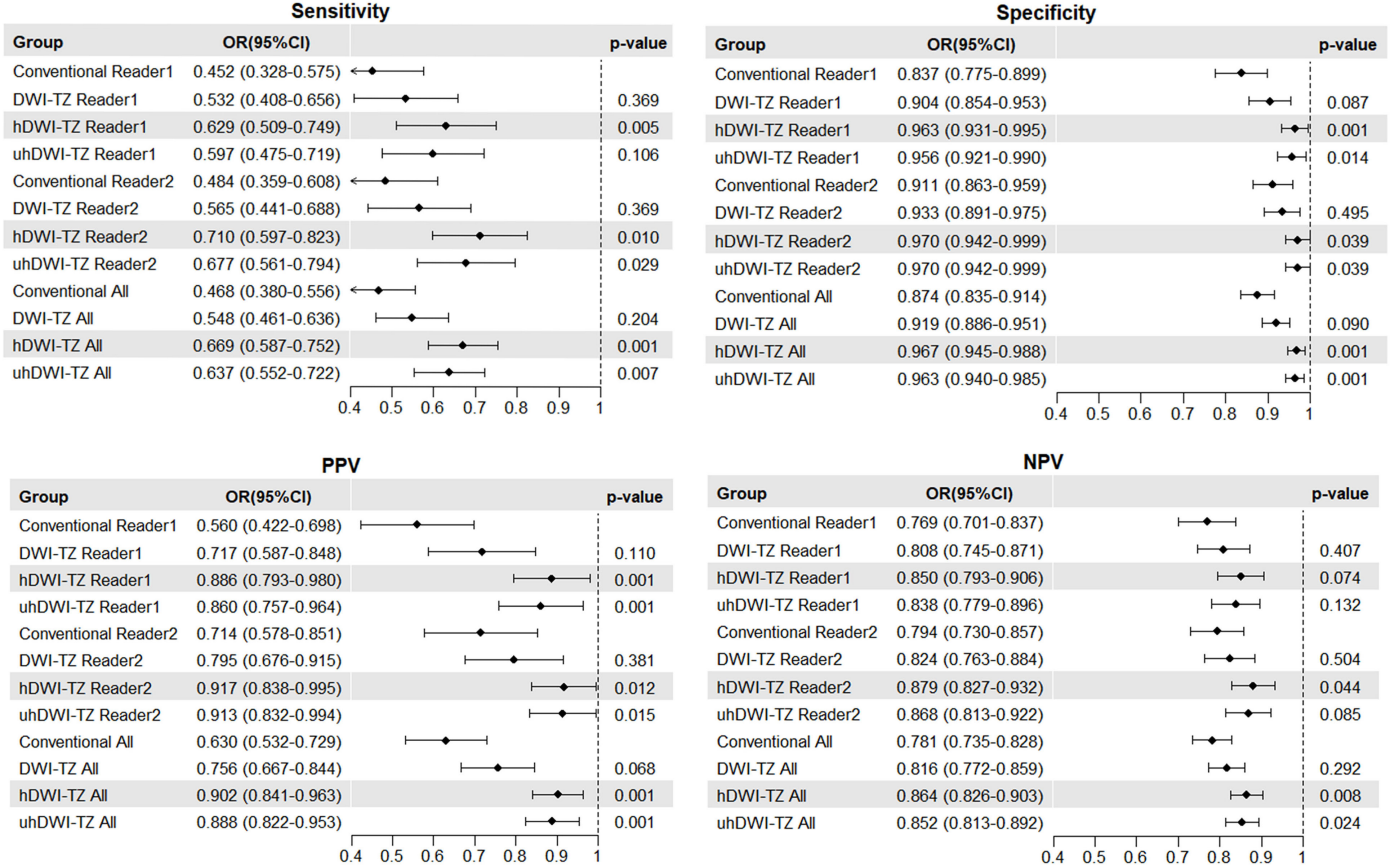
Diagnostic performance of conventional and three different B-value diffusion-weighted imaging-guided TZ scores for PI-RADS category 4 or greater positive lesions.

**Figure 5:**
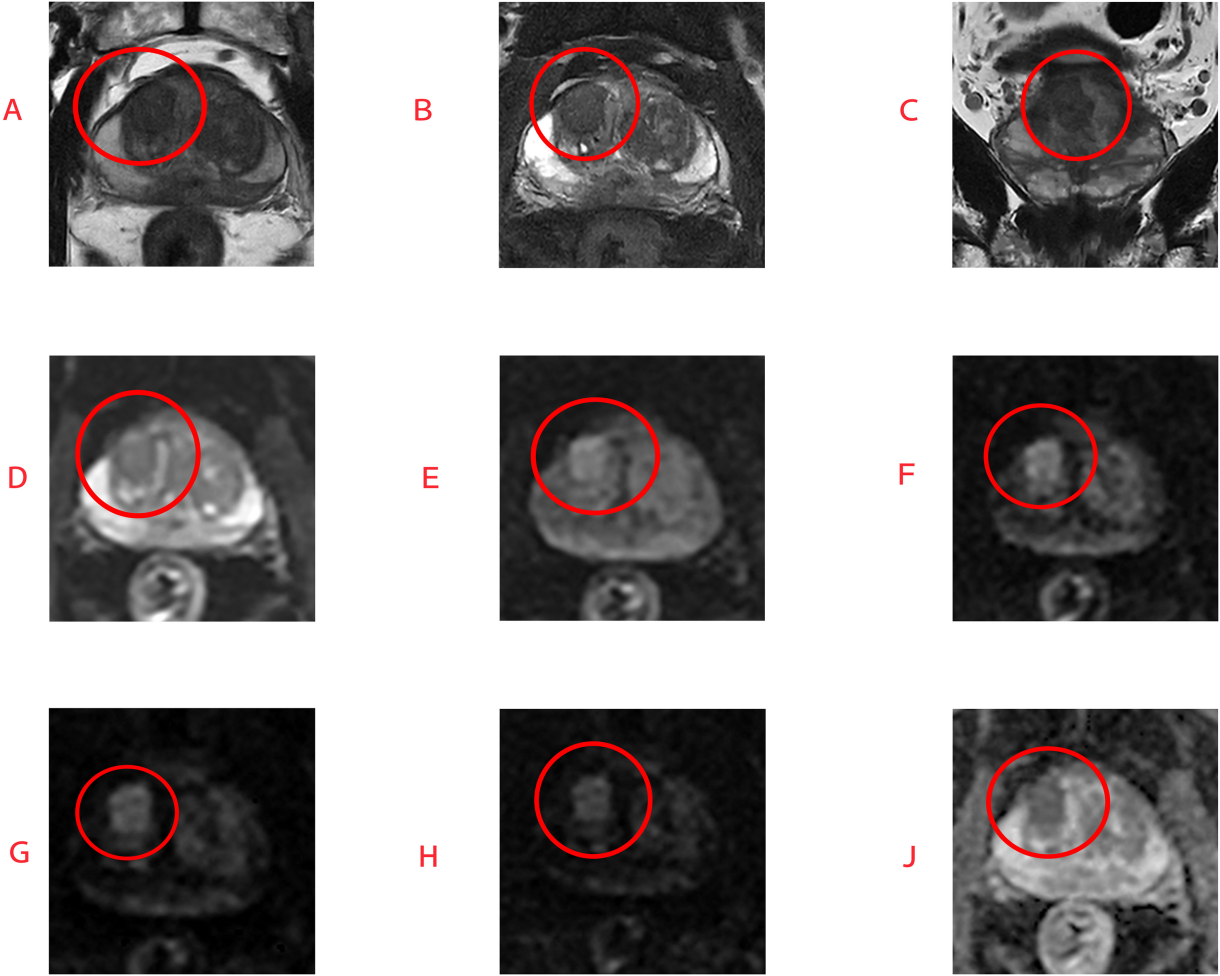
The patient is 65 years old. MRI analysis revealed a lesion scoring three on T2-weighted images **(A–C)** and five on DWI, diffusion-weighted imaging sequences **(D–J)**. Subsequent biopsy pathology results showed a GS, Gleason score of 8, confirming the superior diagnostic value of DWI scoring in prostate cancer assessment.

Furthermore, the F1 scores for h-DWI-TZ and uh-DWI-TZ were significantly higher than those for conventional sequences (0.794 and 0.776 vs. 0.545; p<0.001). When using a stricter threshold of PI-RADS ≥4, h-DWI-TZ and uh-DWI-TZ guided TZ scores remained significantly superior to conventional scoring in sensitivity, specificity, and PPV; In contrast, DWI-TZ scoring showed no significant differences from traditional scoring in sensitivity, specificity, PPV, or NPV. The relevant results are presented in **Figures 4**, **5**.

When using the conventional PI-RADS v2.1 scoring system, senior radiologists (with 11 years of experience) and regular radiologists (with 5 years of experience) identified 39 and 64 false-positive lesions, respectively. However, when employing DWI-guided TZ scoring (PI-RADS ≥3 considered positive) with different b-values, the number of false-positive lesions decreased significantly: h-DWI-TZ group: 13 lesions by senior radiologist, 24 lesions by less experienced radiologist; uh-DWI-TZ group: 14 lesions by senior radiologist, 23 lesions by less experienced radiologist; DWI-TZ group: 25 lesions by senior radiologists, 41 lesions by less experienced radiologists.

## 4 Discussion

This prospective study aims to compare the diagnostic efficacy and interobserver agreement of three different b-value DWI-guided TZ scoring systems versus conventional PI-RADS v2.1 scoring for diagnosing TZPCa. Given the clinical and radiographic overlap between TZPCa and BPH, which often complicates clinical decision-making, enhancing the differential diagnostic capability of TZ lesions holds significant clinical importance.

**Figure 6:**
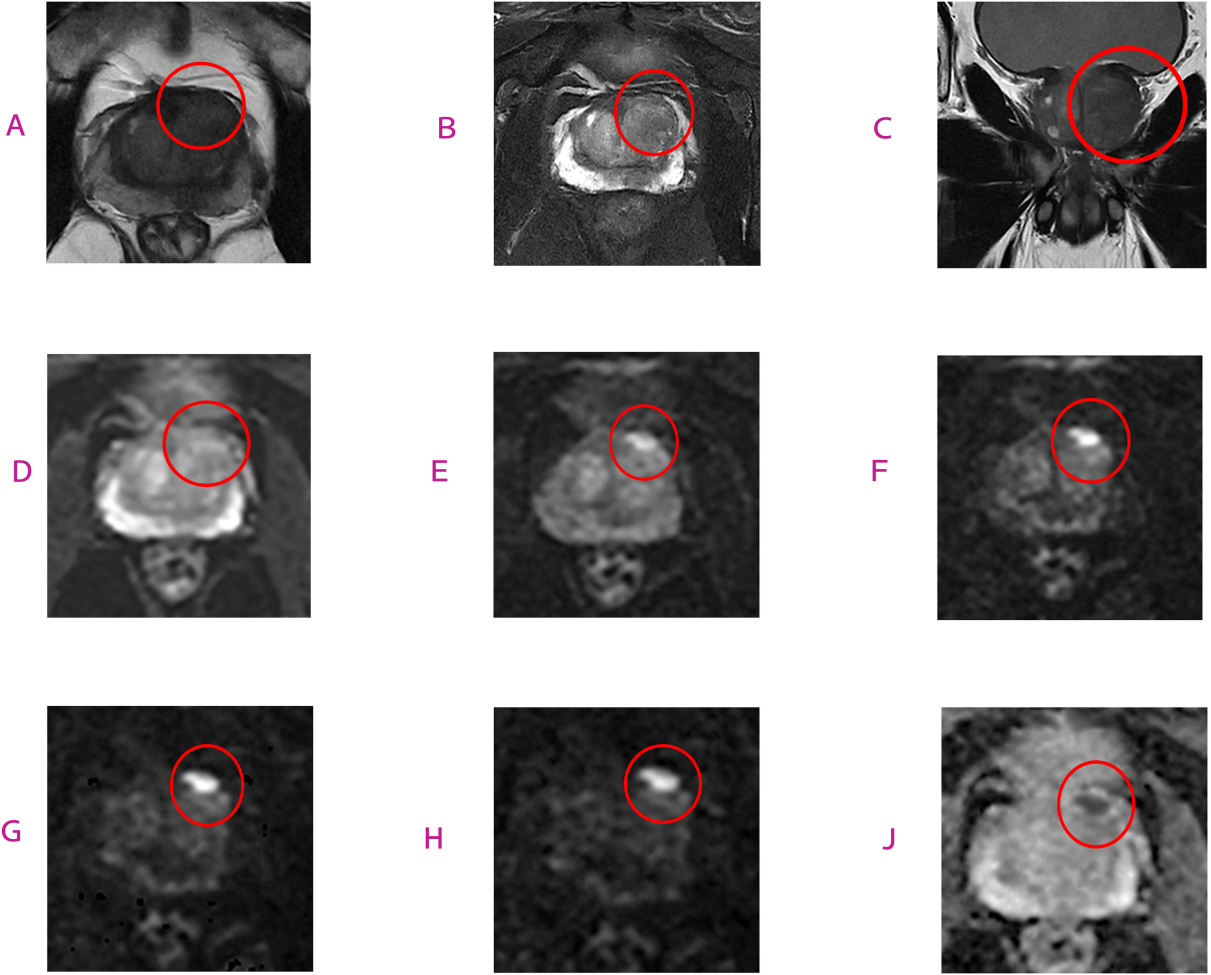
This is an MRI image of a 63-year-old patient with prostate cancer. T2-weighted images **(A–C)** show blurred lesion margins; DWI sequences at b=0 **(D)** and b=800 **(E)** are also shown; however, in the DWI sequences at b=1500 **(F)**, b=2500 **(G)**, and b=3000 **(H)**, as well as the ADC map **(J)**, the lesion (circled in the image) demonstrates distinct areas of diffusion restriction. Final pathological diagnosis confirmed transitional zone prostate cancer with a Gleason score of 9.

**Figure 7:**
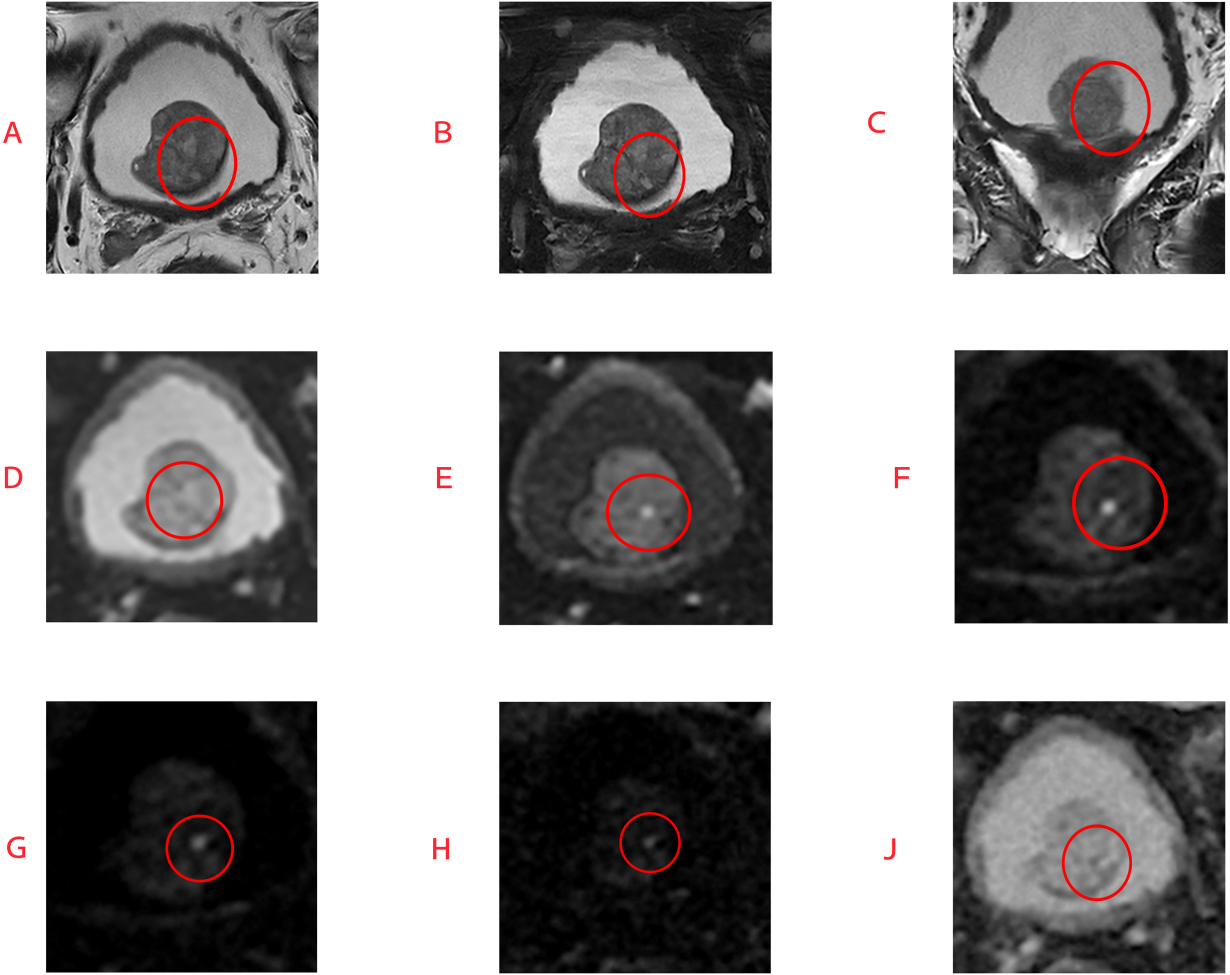
The patient is a 59-year-old male. Prostate MRI images reveal: blurred lesion margins on T2-weighted images **(A–C)**; DWI sequences at b=0 **(D)** and b=800 **(E)** are also shown; however, on DWI sequences with b=1500 **(F)**, b=2500 **(G)**, and b=3000 **(H)**, as well as the ADC map **(J)**, the region (circled in the image) demonstrates distinct diffusion-restricted lesions. Final pathological diagnosis confirmed transitional zone prostate cancer with a Gleason score of 7.

The results of this study indicate that TZ scores based on high b-value (h-DWI-TZ) and ultra-high b-value (uh-DWI-TZ) significantly outperformed conventional PI-RADS v2.1 TZ scores in diagnosing TZPCa, demonstrating superior sensitivity, specificity, and positive predictive value (PPV), with statistically significant differences. Regarding inter-observer agreement, the Kappa values for TZ scores derived from the three b-value-specific DWI sequences were significantly higher than those for conventional scoring (at PI-RADS ≥4: h-DWI-TZ Kappa=0.684 > uh-DWI-TZ 0.650 > DWI 0.544 > conventional PI-RADS v2.1 0.368), suggesting that clinical radiologists may preferentially rely on DWI sequences over T2WI when evaluating TZ lesions (**Figures 6**, **7**).

Rozenkrantz et al. (25) noted that the sensitivity of T2WI alone for detecting TZPCa was only 30%, whereas combining it with DWI (b=2000 s/mm²) increased sensitivity to 56.5–72.2% (a more than 12% increase compared to 50.0–54.8% with b=1000 s/mm²). Kitajima et al. (32) further validated the efficacy of ultra-high b-values (2000 s/mm²), achieving a sensitivity of 80.9% and a specificity of 79.0%. Our findings are highly consistent with these prior conclusions: First, regarding diagnostic performance, the specificity (0.863) of the h-DWI-TZ group aligns with that reported by Lee et al. (17) (0.896). Second, regarding technical advantages, it demonstrated significant improvement over the traditional PI-RADS v2.1 scoring system and also showed major performance enhancements compared to the TZ scoring system, which is based solely on standard DWI with a b-value of 1500.

Prior studies have demonstrated interobserver variability in applying PI-RADS v2.1 to the TZ: Tamada et al. (33) reported a Kappa value of 0.645; Byun et al. (34)reported Kappa values ranging from 0.505 to 0.770 across three scenarios (entire lesion/PI-RADS ≥3/≥4); and Wei et al. (35) reported values from 0.602 to 0.835 across five study groups. Our findings align with this established range of variability, although the interobserver agreement for DWI-guided TZ scoring in our study (Kappa: 0.450–0.689 for threshold ≥3; 0.544–0.684 for threshold ≥4) falls within the lower-to-mid range of the spectrum reported by Wei et al. This slight reduction in concordance may be attributed to two primary factors (1): the relatively limited sample size, which may affect the robustness of reliability estimates; and (2) eddy current effects at ultra-high b-values causing geometric distortion and signal pile-up, which obscure lesion margins and increase subjective interpretation difficult (36).

Unlike the traditional PI-RADS v2.1 scoring system, which requires comprehensive evaluation of numerous features from both T2WI and DWI, the diagnostic logic of TZ scoring guided by high b-value DWI is significantly simplified: DWI sequences can effectively exclude some suspected lesions, with the remaining lesions undergoing multidimensional assessment in combination with T2WI features. Although TZPCa diagnosis based on T2WI features (e.g., lenticular morphology) exhibits high specificity, this approach heavily relies on observer expertise. In contrast, DWI sequences enhance diagnostic standardization by identifying relatively objective features, such as focal low signal on ADC maps and/or high signal on high-b-value DWIs.

It is noteworthy that although the standard b-value (1500 s/mm²) DWI-TZ score did not show statistically significant differences compared to conventional PI-RADS v2.1 scores across most diagnostic indicators, it demonstrated a consistent numerical advantage. This trend suggests that the DWI-TZ score holds greater diagnostic potential. The lack of statistical significance may be attributed to the inadequate contrast between benign and malignant tissues in standard b-value DWI, as well as overlapping ADC values between low-grade and high-grade PCa (37).

Furthermore, although uh-DWI-TZ (b = 3000 s/mm²) provides stronger background signal suppression and theoretically higher contrast-to-noise ratio (CNR), its diagnostic performance proved comparable to or slightly inferior to h-DWI-TZ (b = 2500 s/mm²). This observation highlights the classic trade-off in DWI physics: while increasing the b-value elevates tissue contrast, it simultaneously induces a substantial reduction in signal-to-noise ratio (SNR) and exacerbates geometric distortion and susceptibility artifacts. Consequently, image noise may obscure subtle lesion characteristics or mimic pathological hyperintensity, potentially explaining the slight numerical increase in false-positive findings observed with uh-DWI-TZ (14 for seniors) compared to h-DWI-TZ (13 for seniors).

Crucially, our direct head-to-head comparison revealed no statistically significant difference between b = 2500 and b = 3000. This suggests that the incremental diagnostic benefit of increasing b-values from 2500 to 3000 s/mm² is marginal, and the theoretical gain in contrast at ultra-high b-values appears to be offset by the detrimental effects of low SNR and artifacts. Therefore, higher b-values do not always equate to better diagnostic accuracy, and whether b = 3000 or b = 2500 is more suitable for clinical application remains questionable (38, 39).

Our study employed a DWI-prioritized algorithm (17) for the TZ, wherein lesions not visible on DWI/ADC were not scored, even if detected on T2WI. While this approach theoretically could affect sensitivity for lesions with isolated T2WI visibility, the actual number of excluded lesions in our cohort was small (1–11 across readers and sequences), suggesting a limited quantitative impact on the present results. The protocol’s performance is therefore most directly applicable to DWI-apparent lesions, and its generalizability to lesions visible only on T2WI warrants further investigation.

### 4.1 Limitations

This study has the following limitations: First, the number of cases included remains relatively small. Second, as a single-centre study, differences in patient characteristics and institutional practices may affect the generalizability of the results. Future research should conduct larger-scale, multicenter studies to validate the generalizability of high b-value DWI-guided TZ scoring and explore its potential integration with artificial intelligence algorithms such as deep learning.

### 4.2 Conclusion

This study achieved two key objectives: First, compared with PI-RADS v2.1, DWI-guided TZ scoring improved interobserver agreement and diagnostic performance for TZPCa. Second, when comparing the diagnostic performance of DWI-TZ, h-DWI-TZ, and uh-DWI-TZ, both h-DWI-TZ and uh-DWI-TZ groups demonstrated significantly superior consistency and diagnostic performance compared to the DWI-TZ group. This suggests that high-b-value DWI-guided TZ scoring may hold greater potential for clinical decision support. Future studies should validate these models in larger cohorts and through multicenter external validation.

## Data Availability

The raw data supporting the conclusions of this article will be made available by the authors, without undue reservation.
